# Economy-wide impacts of road transport electrification in the EU

**DOI:** 10.1016/j.techfore.2022.121803

**Published:** 2022-09

**Authors:** Marie Tamba, Jette Krause, Matthias Weitzel, Raileanu Ioan, Louison Duboz, Monica Grosso, Toon Vandyck

**Affiliations:** a European Commission, Joint Research Centre (JRC), Seville, Spain; b European Commission, Joint Research Centre (JRC), Ispra, Italy; c Independent Researcher, Milan, Italy

**Keywords:** Economic modelling, Electric vehicles, Road transport, Climate mitigation, Employment

## Abstract

While electrification of road transport is a key component of decarbonisation, the implications for the broader economy and related jobs remain underexplored. We quantify these impacts in the EU in a global Computable General Equilibrium (CGE) model, combining techno-economic assumptions about electric vehicles with deployment scenarios derived by energy models. We augment input-output tables underlying the JRC-GEM-E3 model with an explicit representation of vehicle manufacturing and upgrade the modelling of vehicle purchase and operation. Our findings illustrate that greater road transport electrification reduces the overall costs of climate mitigation, primarily driven by lower fuel costs for electric vehicles and a faster decline of battery costs. Transport electrification alters supply-chains and leads to structural shifts in employment from traditional vehicle manufacturing towards battery production, electricity supply and related investments. Finally, we expand the set of labour market indicators to cover skills and occupations, to refine the socio-economic assessments of climate policy.

## Highlights

• We model the macroeconomic impacts of road transport electrification in the EU.
• Decomposed impacts of electric vehicles' techno-economic characteristics on costs.
• Higher electrification can lower the costs of climate mitigation, increasing employment.
• New model framework includes labour market projections on skills and occupations.

## 1 Introduction

Global CO_2_ emissions from transport have continued to rise in recent years across all modes (IEA, 2020a). In the EU, transport greenhouse gas (GHG) emissions were 33 % higher in 2019 compared to 1990 levels, while other sectors achieved emissions reductions over the same period (e.g. energy supply −39 %, industry −35 %, EEA, 2020). This is in stark contrast with the EU's objectives of 55 % emission reductions by 2030 compared to 1990 and to achieve net zero emissions by 2050.^1^

Road transport, accounting for nearly one-fifth of total EU GHG emissions, has therefore become a priority sector for climate action and a key element of the European Green Deal (European Commission, 2019). To encourage rapid emissions reductions in road transport, Europe has recently proposed new initiatives for stronger emissions standards for cars and vans, the set-up of a new emission trading scheme for buildings and road transport, as well as new regulation for alternative fuel and charging infrastructure (European Commission, 2021, European Commission, 2021).

Numerous studies have explored scenarios of transport decarbonisation, its role in global climate change mitigation strategies, and the potential contributions of different technologies, in particular electric vehicles (EVs). McCollum et al. (2014) highlight that transport electrification can lead to lower cost decarbonisation pathways than reliance on alternative liquid fuels (biofuels and synthetic fuels). Similarly, Rottoli et al. (2021) find that direct electrification is a better option for decarbonisation of the light-duty vehicle fleet in Europe compared to fuel-cell or synthetic fuel options, as it significantly reduces the primary energy demand required to meet the needs of the transport system. Zhang and Fujimori (2020) explore the transport-energy supply integration, stressing how a stringent electrification of transport with decarbonisation of electricity supply could lead to lower mitigation costs than alternative scenarios.

Such findings are repeatedly confirmed across modelling methodologies for long-term climate mitigation scenarios, although to different degrees. Edelenbosch et al. (2017) review findings from eleven Integrated-Assessment-Models (IAMs) and report that CO_2_ emission reductions in mitigation scenarios are mainly achieved through fuel switching (to electricity and hydrogen) and fuel efficiency gains. Yeh et al. (2017) in a comparison of IAMs and transport-focused models find that the latter group tend to place a greater emphasis on modal shifts and efficiency improvements. Pietzcker et al. (2014) compare scenarios from five “energy-economy” models' projections to confirm that the responsiveness of the transport sector to climate policy depends on the technology options available within the energy system in the model.

EVs in particular have become a clear-focus in long-term modelling of road transport decarbonisation. Ghandi and Paltsev (2020) integrate a total cost of ownership model for various types of light-duty electric vehicles in the Emissions Prediction and Policy Analysis (EPPA) CGE model to project global stocks of EVs to 2050. They show that the global share of EVs in the light-duty vehicle fleet could reach as high as 50 % in 2050 in ambitious climate scenarios. The BP energy outlook (BP, 2020) presents a BAU scenario where EVs account for 30 % of four-wheeled vehicle kilometres travelled in 2050, whereas in the “Rapid” scenario they cover 70 % and in the “Net Zero” scenario 80 % of 2050 activity at global level. Electrification of the vehicle fleet concerns mostly the segments of two and three wheelers, passenger cars and light and medium-duty trucks. BNEF (2021) present an Economic Transition Scenario, in which EVs make up 73 % of 2050 global vehicle sales, and 54 % of the global passenger car fleet.

For Europe, Karkatsoulis et al. (2017) using a transport-augmented energy system model find that plug-in hybrid electric vehicles (PHEV) and EVs can represent over 70 % of the passenger car fleet by 2050 under a decarbonisation policies scenario. Krause et al. (2020) explore the CO_2_ reductions implications of expert-based scenarios for ambitious EV penetration (64–85 % of vehicle activity covered by PHEVs and EVs by 2050). In a study by Ricardo on behalf of Concawe (2018), different European light duty vehicle fleet and fuel options are considered, including a High EV scenario which assumes a 90 % share of battery electric vehicles (BEV) in the 2050 vehicle fleet, and a low carbon fuels scenario which proposes biofuels and e-fuels as a primary means of emission reduction, combined with a fleet containing 47 % of PHEV, BEV and fuel cell EV by 2050.

Despite the remaining uncertainty in terms of market shares and penetration of EVs, large-scale electrification of the fleet (alongside a decarbonised electricity grid) is now considered a viable option to curb the sector's emissions (Alonso Raposo et al., 2019). While relatively small today, the global stock of EVs has been growing rapidly in recent years. Global sales of EVs have increased by more than 60 % per year over the last decade, and there is evidence that EVs will not be as affected by the global slump in car sales from the COVID-19 crisis (IEA, 2020b) compared to conventional models. In addition, recovery spending may be targeted at subsidizing EV purchases or charging infrastructure.

To satisfy this new demand for EVs, almost all major auto-manufacturers have announced significant investments in EVs production lines and hundreds of new models are expected to enter the market in the coming five years (IEA, 2020c; McKinsey, 2020). The sector and its supply chain are already experiencing substantial change, warranting much interest from industry groups and policy-makers, given its role as major employer. Bottom-up, country- or region-focused studies can provide some insights in the sectoral economic and employment impact of such transitions in the medium-term. For instance, a study on the German auto-manufacturing sector (Frauhofer, 2018) finds that electrifying the fleet could translate into absolute job losses within the sector of between 11 % and 35 % by 2030, depending on the type of power train technology, their adoption trajectories and productivity gains from industrial digitization and automation, requiring a rapid transition towards new skills and qualifications. The European Association of Electrical Contractors (AIE, 2020) finds that the potential job creation in the electricity value chain from electro-mobility (charging points operation and maintenance, electricity generation, grid reinforcement and battery manufacturing) could outweigh the potential job losses in auto-manufacturing.

While sectoral studies provide useful insights on the evolution of auto-manufacturing and its key suppliers, the peer-reviewed literature on broader economic and employment implications of a large-scale road transport electrification remains remarkably scarce. Yet, the potential breadth of economic consequences could extend well beyond the vehicle-manufacturing sector alone. For example, EVs need inputs from battery manufacturing. In addition, moving to EVs will also disrupt fuel supply chains and affect electricity demand. The transition will impact competitiveness, trade balances, costs for providers of transport services, vehicle maintenance requirements as well as the labour market. Furthermore, this transformation is expected to span several decades with potential periods of transition where investments in new technologies and infrastructure could be front-loaded. Accordingly, the issue is best tackled in a dynamic economy-wide framework, which can capture both the direct and wider economic impacts of a shift to EVs in road transport, as well as the medium and long-term dimensions.

While previous studies have used such methodology to explore the macroeconomic implications of climate policy pathways in general (see for example Weitzel et al., 2019; Vrontisi et al., 2020), few have focused specifically on the contribution of transport. Karkatsoulis et al. (2017) combines projections from a transport-energy system model (PRIMES-TREMOVE) with a global Computable General Equilibrium (GEM-E3) to explore CO_2_ reductions pathways in the European transport sector in general, but they do not address the specific consequences of electrification of road transport, and its supply chain and employment impacts. Ghandi and Paltsev (2020) also adopt an economy-wide modelling framework for road transport electrification, but their work focuses on identifying EV penetration pathways for light-duty vehicles, and do not place emphasis on their wider economic implications.

This paper assesses the macroeconomic impacts of road transport electrification in the European Union in the context of climate neutrality, and the transformation it implies across the economy and the labour market. We propose a comprehensive modelling approach, which combines engineering-based assumptions on vehicle manufacturing and operation, a global computable general equilibrium model and long-term projections on occupation and skills. Our work is novel in three main ways:

First, our focus on road transport electrification, rather than a wider look at decarbonisation policies and technologies, enables an in-depth study of the corresponding issues. We extend the model and develop a set of scenarios particularly for this purpose. The scenarios share a common climate mitigation goal but differ in the degree of road transport electrification, which enables the analysis of macroeconomic and employment impacts directly attributable to road transport electrification.

Second, through a decomposition analysis, we identify and quantify several impact channels of moving from conventional oil-fuelled internal combustion engine vehicles to EVs, from the manufacturing of components to the operation of vehicles.

Finally, we couple the macroeconomic model results on sectoral employment changes with a complementary dataset on the labour force to determine the impacts of the EV transition on occupations and skill composition. These additional indicators enrich the analysis of consequences for the EU workforce and could help informing policies that seek to align labour demand with supply.

Section 2 describes the analytical framework used in the paper, including a description of the CGE model (JRC-GEM-E3), its extensions specific to road transport electrification, the techno-economic assumptions behind the electrification scenarios, and the use of labour force data and projections. Section 3 presents the results of the scenarios and decomposition analysis. Section 4 discusses the implications of the results for policy-makers and concludes.

## 2 Methods

### 2.1 The model

JRC-GEM-E3 (General Equilibrium Model for Economy-Energy-Environment) is a recursive dynamic CGE model of the world economy.^2^ The version used in this study covers the European Union, alongside 13 other major countries and regions. With a detailed sectoral disaggregation of energy activities, as well as endogenous mechanisms to meet carbon emission constraints, JRC-GEM-E3 has been extensively used for the economic analysis of climate and energy policy impacts (see Keramidas et al., 2020 for global mitigation scenarios or European Commission (2020a) for an EU-focussed assessment of 2030 climate targets). The version used in this paper is based on the GTAP database v9.2 (Aguiar et al., 2016).

Firms are cost-minimizing under constant elasticity of substitution production functions and are divided into 33 sectors of activity, with an emphasis on energy intensive industries (e.g. iron and steel, chemicals) and energy production and supply (including 8 electricity generation sectors, 1 electricity distribution and transmission sector, 3 fossil fuel production sectors). The list of sectors of activities in the model is provided in Appendix A.

The representation of transport in the model reflects the aggregation in the GTAP database, which forms the basis for the model structure in the base year. In particular, three transport sectors are represented (encompassing all transport modes), namely air, water and land transport. Road transport is part of the land transport sector, which is both an input to production for firms and a consumption good for households (which can also perform their own transport activity through the purchase and operation of private cars).

For the present modelling exercise, the manufacturing of transport equipment (a single sector of activity in the standard JRC-GEM-E3 model set-up), is split into three new sectors. The manufacturing of conventional motor vehicles sector is separated from the manufacture of other transport equipment^3^ (e.g. airplanes, ships, trains) as proposed by GTAP. We introduce a new sector of activity to represent the manufacturing of electric vehicles (see Section 2.2.). All sectors are interlinked by providing goods and services as intermediate inputs (and investment inputs) to other sectors, used in combination with production factors (labour, skilled or unskilled, and capital).

Households are the owners of the production factors and receive income, which they use to maximize utility through consumption. Household consumption is split between 14 different consumption categories. A distinction is made between durable goods (heating and cooking equipment, private vehicles) and non-durable goods. Durable goods can be used through the consumption of linked non-durable goods (for example fuels for heating or transport, maintenance expenditures). Consumption categories are linked to the 33 products of industrial sectors through exogenously defined consumption matrices.

Government is considered exogenous, while bilateral trade-flows are allowed between countries and regions. In 5-year steps, from 2015 out to 2050, equilibrium is achieved at the global level on the goods and services markets and for factors of production, through adjustments in prices. Two labour market closures are available, the first, representing a long-term perspective of fixed unemployment, and the second where employment responds to changes in wages. Results of both options are presented below.

### 2.2 Manufacturing of battery electric vehicles

In order to reflect differences in the production processes of battery electric vehicles (BEVs) and internal combustion engine (ICE) vehicles, we enhance the base year input-output data from GTAP with techno-economic data on the components of EVs. We introduce a new sector, representing the manufacturing of BEVs,^4^ which does not exist in the GTAP base year data (2011). We define its production structure by modifying the structure of conventional motor vehicle manufacturing in the EU27 + UK^5^ to reflect that conventional vehicles and EV have a similar vehicle body, but a shift in composition (in value) is assumed from the removal of the combustion engine towards the purchase of a battery. We compute the production structure of EVs assuming that the difference in total production costs corresponds to the difference average pre-tax price. Pre-tax retail prices for comparable ICE and BEV cars are taken from BNEF (2017) for cars, converted to euros. The same source also specifies the pre-tax price of an average 2016 new BEV of the same segment. For vans, as no generic data was available for equivalent ICE and BEV models, prices are based on Renault's suggested pre-tax retail prices for the Renault Kangoo Express^6^ (conventional, 16,200 EUR) and Kangoo Z.E.^7^ (electric, 29,900 EUR) in 2018.

Pre-tax retail prices for comparable ICE and BEV trucks are taken from Sen et al. (2017). In terms of sectoral input composition, we furthermore make three assumptions. First, the production of a BEV needs only 75 % of the input from the manufacturing sector of an equivalent conventional car (Cuenca et al., 2000) as it does not need an engine but a simpler electric motor, no gearbox, and has less costly transmission. Second, the battery cost of the BEV is calculated as the pre-tax price difference versus a conventional car. Third, the contribution of all other sectors, as well as the value added, is the same as for an equivalent conventional vehicle in value.

Weighted by the shares of vehicle segments in the fleet,^8^ we compute the input structure of the new electric vehicle manufacturing sector. Table 1 presents the resulting production input structure for light duty vehicles in shares for both sectors. The share of inputs in percentage terms for BEVs is therefore a result of the shifts in inputs assumed above and the higher value of one unit of production (i.e. the cost difference between a conventional and a BEV. While manufacturing of conventional vehicles relies most heavily on own inputs (capturing chassis and engine components and parts), approximately 50 % of the intermediate input costs for BEVs are attributable to the battery costs, included in the other equipment goods sector.

**Table 1:** Input structure of manufacturing of motor vehicles. Input shares (percent).

	Manufacture of conventional vehicles	Manufacture of electric vehicles
Manufacture of conventional vehicles	28.3	0.0
Manufacture of electric vehicles	0.0	13.2
Market services	14.7	9.2
Other Equipment Goods^a^	14.1	50.8
Non-ferrous metals	8.5	5.3
Chemical products	6.5	4.0
Ferrous metals	3.4	2.1
Land transport	2.0	1.3
Consumer goods industries	1.0	0.6
Electric goods	0.8	0.5
Non-metallic minerals	1.5	0.9
Others (< less than 1 % each)	2.6	1.6
Value added	16.7	10.4

a Sector “Other equipment goods includes the production of batteries” (corresponding to NACE Rev.2 C27 (Documentation on NACE Rev. 2 Classification of economic activities in the European Community available at: https://ec.europa.eu/eurostat/documents/3859598/5902521/KS-RA-07-015-EN.PDF)).

### 2.3 Modelling scenarios of electric vehicle deployment

We consider the two vehicle types (ICE and EVs),^9^ and households and firms move gradually from conventional towards EVs over the projection period. We compare a baseline future with three alternative scenarios of low, medium and high levels of transport electrification.

For these four alternatives, we use projections of the vehicle fleet and fuel consumption out to 2050 from energy system models: a baseline, reflecting climate and energy policies agreed by the EU as of June 2018, and three scenarios aiming for the “well below 2°C aim”, or GHG emissions reduction levels in 2050 of around 80 % compared to 1990 target. For the EU, fleet and energy consumption projections are based on scenarios from the PRIMES model developed for the in-depth analysis accompanying the EU's 2050 long-term strategy (European Commission, 2018). Projections from the PRIMES' ELEC scenario are used in our high-electrification scenario, while the projections from the H2 scenario are used in the case of low electrification. In the absence of a direct scenario available in the same analysis for our medium-electrification case, intermediate projections were derived using averages between the two extreme scenarios. Information about the respective PRIMES scenarios and underlying technological and policy assumptions is provided in the in-depth analysis (European Commission, 2018, Table 1 p. 56). For non-EU countries and regions, which enable us to study issues such as international competitiveness, we use low, medium and high electrification scenario projections, as well as emission reduction targets from the POLES-JRC model produced for the Global Energy and Climate Outlook 2019 (Keramidas et al., 2020).

We impose the penetration of EVs in both the private car fleet for households and the fleet of other road vehicles (trucks, buses and passenger light-duty vehicles) for firms. Additionally, the corresponding fuel mix trajectories from the same models and scenarios are used for households and firms. A summary of transport electrification assumptions for the year 2050 across scenarios for the EU is provided in Table 2.

**Table 2:** Road transport electrification indicators in EU27+UK in 2050.

	Baseline	Low Electrification	Medium Electrification	High Electrification
Share of EVs in the road transport fleet (number of vehicles)				
Private transport	44.9 %	59.5 %	65.0 %	70.5 %
Freight and public transport	28.7 %	21.2 %	36.1 %	50.9 %

Share of electricity in the road transport final energy consumption (%)				
Private transport	23.0 %	52.6 %	62.3 %	72.1 %
Freight and public transport	8.3 %	7.6 %	25.9 %	44.3 %

Source: European Commission, 2018

The high electrification scenario reaches a very high level of EV penetration in the fleet by 2050, with over 70 % for private cars and 50 % for other vehicles. In contrast, in the low electrification scenario, the EV share is lower than in the baseline for freight and public transport vehicles, reflecting the deployment of alternative low-carbon technologies (e.g. biofuels). These assumptions therefore represent a broad range of potential EV penetration outcomes, reflecting the uncertainty involved.

We implement these exogenous shifts in fleet and energy consumption through time-dependent households' consumption matrices (which link consumption categories to sectors of activity in the model) and firms' investment matrices (which link branches of activity to investment expenditures). For households, we use the share of EVs in new car purchases to update the composition of the household consumption category “purchase of new vehicle” over time: the share of manufacturing of conventional vehicle (sector 32 in Appendix A) is progressively shifted to the manufacturing of EVs (sector 33). The consumption matrix is also adjusted to reflect the evolution of the fuel mix towards electrification, alongside general fuel efficiency improvement to capture the change in total fuel consumption per vehicle kilometre over time. We do not impose an exogenous evolution of the consumption matrix in other consumption categories, such as the use of public transportation, and as such, we do not assume exogenous modal shifts resulting of electrification from energy system models.^10^

A similar approach to households is applied for the land transport sector using the investment matrix: transport firms' investments in conventional vehicles are progressively replaced by investments in EVs to reflect the shares in the table. We also exogenously assume the fuel mix from energy models using the share of each fuel (oil, biomass, and electricity) in total intermediate inputs in the land transport sector. Finally, we do not consider the investments required in charging infrastructure, nor their manufacturing and maintenance, and choose to focus on the deployment of electric vehicles in the fleet.

### 2.4 Battery costs, learning effects and maintenance costs

While the relatively higher cost of EVs compared to their equivalent internal combustion engine vehicle options remains a key barrier to adoption (Rezvani et al., 2015), battery costs are widely expected to fall sharply with accelerating adoption of the technology. For example, the costs of li-ion battery packs (the most prominent battery technology in EV sales worldwide to date) are expected to fall rapidly, with learning rates of approximately 16 % (unit cost reductions for each doubling of production capacity), based on a recent literature review (Tsiropoulos et al., 2018). Recent analysis by BNEF^11^ and UBS^12^ suggests that falling battery costs could result in price parity between conventional and electric vehicles as early as 2025. Therefore, in the model, the deployment of EVs is associated with an initial cost differential with conventional ICE vehicles, which reduces over time with battery cost reductions, through exogenously defined learning curves, consistent with deployment trajectories from the energy models. For the analysis, we impose a cost reduction trajectory for batteries in EVs consistent with the learning-by-doing assumed in the EU's Long-term Climate strategy (European Commission, 2018) to remain consistent with the deployment and fuel consumption scenarios. These assumptions can be found in Hill et al., (2016, Table 2.4, p. 9, central, low and high estimates).

Furthermore, the literature suggests that battery electric vehicle have lower maintenance costs than comparable conventional vehicles, attributed to various factors: fewer moving parts (Palmer et al., 2018; Logtenberg et al., 2018; Mitropoulos et al., 2017; Lebeau et al., 2013; Feng and Figliozzi, 2012); no need for changing oil and filters (Moon and Lee, 2019; Logtenberg et al., 2018; Lebeau et al., 2013); and regenerative braking systems (Palmer et al., 2018; Logtenberg et al., 2018; Hoekstra et al., 2017; Lebeau et al., 2013). The order of magnitude of maintenance cost reduction for BEV versus conventional vehicles varies substantially among different studies, and depends on the type of vehicles considered, their sizes, mileages covered, geographical locations, technical and mechanical characteristics. Our review of the literature (Keramidas et al., 2020, Table 18) illustrates the wide spread of findings, ranging from a minimum reduction of 16 % (Gnann et al., 2014) to a maximum of 75 % (Lee et al., 2013). In the model, an average value of 30 % of maintenance cost reduction of BEV compared to conventional vehicles is used, applied as a reduced expenditure on maintenance services by owners of BEVs in both passenger and freight transport.

### 2.5 Employment dynamics: occupations and skills

In order to further study the impacts on the European labour market, we decompose the employment results from the JRC-GEM-E3 model into impacts on occupations and skills. We do so by linking the CGE sectoral employment results to projections of labour market dynamics on occupations and skills – where occupation is defined as a set of jobs with similar tasks and duties, and skill levels refers to the level of education or on-the-job training required to perform said tasks and duties (ILO, 2012). For this purpose, we use CEDEFOP's latest forecasts on the occupational structure by sectors and on the composition of skills at the job level (CEDEFOP, 2020). These projections aim to reflect expected structural changes within the economy (i.e. economic growth and changes in its sectoral composition), as well as in occupational and skill structure due to factors such as digitization and automation. The dataset includes projections on the occupational and skill breakdown (41 occupations in 9 occupational groups and 3 skill levels, see Appendix C) for 66 economic sectors out to 2030. We perform a matching and aggregation of sectors using the NACE Rev.2 classification at the 2-digit level to reconcile the sectoral coverage with the 33 economic sectors in the CGE model, resulting in detailed projections for 20 sectors of activity.

The CEDEFOP projections must be augmented to incorporate occupations and skills projections for the new sector introduced in JRC-GEM-E3, which was not present in the statistics, namely manufacturing of BEVs. No quantitative estimates of the occupational or skill structure currently exists for this sector, but a number of studies have identified high-level trends which could impact the manufacturing of vehicles when production lines are adapted towards electric vehicles, such as an increasing need for highly-skilled R&D engineers and technicians working on electronics and IT (Eurofound, 2017).

Based on a simplified text analysis method, we systematically translate the qualitative insights in the existing literature into a set of quantitative assumptions about future occupation and skill evolution in EV manufacturing. We match the qualitative statements on the evolution of jobs and types of skills in Eurofound (2017) to a matrix of 41 occupations used by CEDEFOP. We associate each statement with its implications on whether it suggests increasing or decreasing importance of an occupation within EV manufacturing, and/or an upskilling or deskilling^13^ within a given occupation. The results of this matching exercise is a set of count indicators, summarised in Table 3.

**Table 3:** Expected changes in vehicle manufacturing labour force with increasing EV penetration.

	Occupation share within the sector (number of times mentioned)		Skill evolution within occupation (number of times mentioned)	
	Increase	Reduce	Upskill	Deskill
Science and engineering professionals	7	2	6	0
Information and communications technology professionals	3	0	1	0
Science and engineering associate professionals	2	2	2	0
Information and communications technicians	1	0	2	0
Metal, machinery and related trades workers	0	3	0	0
Electrical and electronic trades workers	3	0	0	0
Stationary plant and machine operators	0	2	0	0
Assemblers	0	4	1	0

Source: Results of text analysis on Eurofound (2017).

Using these count indicators, we modify the projections of occupational share and skill distribution for the traditional manufacturing of motor vehicles from the CEDEFOP Skills Forecasts, to derive the structure of the new BEV sector. One key assumption is that the CEDEFOP forecast for the manufacturing of vehicles captures a “baseline” vision of the sector, with only a limited penetration of EVs, which is consistent with their underlying methodology.^14^

**Fig. 1:**
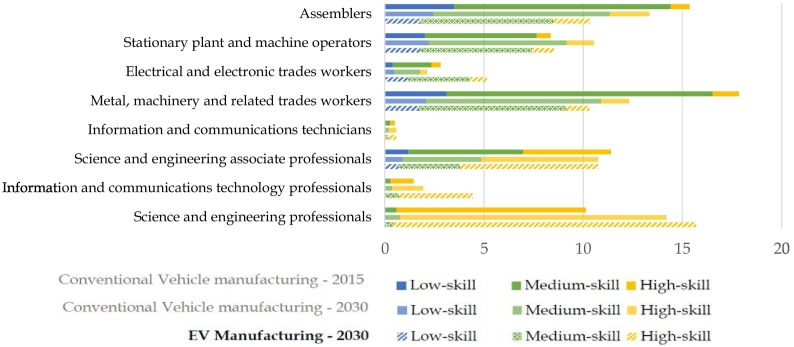
Share of key occupations in Manufacturing of vehicles in 2015 and 2030 (%).

The translation from count indicator to percentage point change in occupation and skill shares in 2030 is further informed by two additional considerations from the original CEDEFOP forecast, which are used as higher and lower bounds for the changes: the existing low-medium-high skill share by occupation in traditional car manufacturing, and the minimum and maximum change in occupational share and skill distribution between 2015 and 2030 across all sectors. Hence, the assumed evolution of the BEV manufacturing sector in terms of occupations and skills remains quantitatively consistent with the existing CEDEFOP forecast. Finally, the translation is also informed by further reading of the wider literature on the evolution of labour demand in vehicle manufacturing in Europe (Eurofound, 2018; Cedefop, 2021; European Sector Skills Council, 2016). The results of the updated Skills Forecasts for manufacturing of BEVs using the above methods is provided in Fig. 1, which compares it with the evolution of shares and skill distributions of the 8 key occupations between 2015 and 2030 in the CEDEFOP forecast for manufacturing of conventional vehicles.

For the majority of occupations, the new BEV assumptions exacerbate the trends assumed in the CEDEFOP forecast for the sector. For Assemblers, and Metal and related trades workers, we assume a deeper decline in the share of these two occupations in the BEV sector's workforce between 2015 and 2030 than the CEDEFOP baseline forecast for manufacturing of vehicles. Information and Communication professionals, and Science and Engineering Professionals, are further reinforced in their projected increased share and upskilling than in conventional vehicle manufacturing. For associate professionals categories (IT technicians and science and engineering associate professionals) our assumptions simply reinforce the upskilling within occupation. In contrast, for two occupations, the results of the text analysis either moderate or invert the trend projected by the CEDEFOP forecast: where stationary plant and machine operators were expected to gain more than two percentage points in their share of total vehicle manufacturing sector employment, they remain stable over time for BEV manufacturing. Finally, electrical and electronic trade workers, which were projected to become a less important occupation in manufacturing of vehicles by CEDEFOP, gain in share of sectoral employment.

We apply this augmented Skills Forecast to the sectoral employment results from the JRC-GEM-E3 model, to obtain sector-occupation-skill projections for the baseline and for each of the three transport electrification scenarios. With these fixed CEDEFOP projections of skills and occupations per sector, any shifts observed in occupations and skills compared to the baseline can be attributed to sectoral employment shifts within the EU27 + UK from the electrification of road transport.

## 3 Results and discussion

In this section, we present the results of modelling the deployment of EVs in the JRC-GEM-E3 model. As described above, the low, medium and high electrification scenarios are modelled over the period 2020–2050 and are presented against a baseline scenario, incorporating a limited ambition for emissions reductions. Results of the electrification scenarios must be interpreted in the context of ambitious climate action of 80 % GHG emission reductions in 2050 compared to 1990.

### 3.1 Macroeconomic impacts

**Fig. 2:**
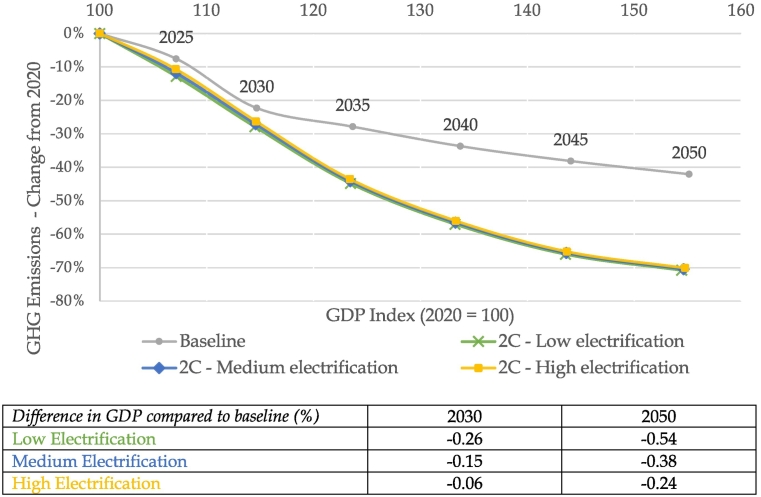
EU Economy and GHG Emission evolution across baseline and scenarios. Source: JRC-GEM-E3 results.

In order to reach the climate targets, GDP growth is strongly decoupled from GHG emissions in all three electrification scenarios. Fig. 2 shows the evolution of GHG emissions and GDP over the modelling period across all scenarios for the EU27 + UK. While the electrification scenarios exhibit similar economic growth pathways as the baseline (more than 1.4 % average annual growth across baseline and all three scenarios), emissions are significantly reduced, indicating a strong shift to cleaner technologies and energy sources.

The costs of climate action, represented as relative change in GDP in the electrification scenarios against the baseline, are shown in the bottom table of Fig. 2. GDP reductions in 2050 from climate action range from −0.24 % to −0.54 %, which indicate a small cost of climate action in GDP terms. This result encompasses the impact of road vehicle electrification, as well as other decarbonisation actions, and in particular the shift towards renewable energy sources in electricity production. In other countries and world regions, climate action scenarios lead to slightly larger GDP losses compared to the baseline than in the EU as shown in previous studies (e.g. Vandyck et al., 2016).

This result of small GDP loss is in line with findings in previous studies on the macroeconomic impact of decarbonisation scenarios in Europe. Using CGE models, Lewney et al. (2018) find a 0.5 % reduction in EU GDP from a 2C scenario compared to a baseline, while Capros et al. (2014) find a 0.6 % reduction in a scenario achieving the 450 ppm stabilisation target (equivalent to 2°). Keramidas et al. (2018) find global GDP reductions between 0.4 and 1.3 % globally across 2C and 1.5C scenarios compared to a baseline. Focusing on deep transport decarbonisation, Karkatsoulis et al. (2017) also find small negative GDP impacts for the EU of increasing the uptake of both electric vehicles and biofuels.

The design of the three scenarios in the current paper allows for further investigation of the benefits of increased electrification of road transport, in the context of climate policies. The scenario with the highest penetration of EVs in the fleet and electricity use in the fuel mix leads to the lowest reduction in GDP compared to the baseline in 2050. In contrast, reaching a climate target with limited road transport electrification leads to the largest reduction in GDP compared to the baseline as further efforts are needed in other sectors with potentially higher abatement costs (e.g. energy intensive industries). This result also holds in other world regions and is in line with previous studies, e.g. Capros et al. (2014) find that a climate mitigation scenario of delayed transport electrification results in higher GDP losses compared to a scenario where transport decarbonises quickly.

### 3.2 Decomposition of impacts

Using the new representation of electric vehicles manufacturing, we can go one step further than existing studies in explaining the above macroeconomic results. As described in Section 2, we exogenously impose the deployment of EVs in the three climate scenarios (low, medium and high penetration respectively). In addition, we introduce a number of assumptions about the manufacturing process and operation of EVs, linked to deployment, based on a review of the literature. In this section, we decompose these various impact channels on the macroeconomic results.

**Fig. 3:**
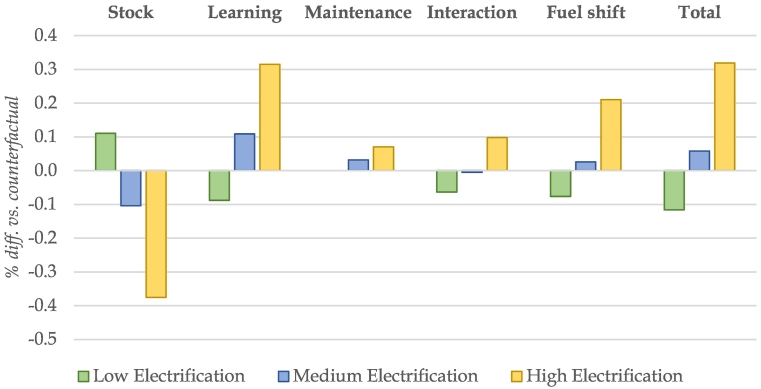
Main impact channels of EV deployment in EU27 + UK GDP results in 2050. Source: JRC-GEM-E3 results.

To obtain the decomposed results, the JRC-GEM-E3 model is run over a number of stylised scenarios incorporating each of the assumptions separately, both under the baseline and electrification assumptions in terms of EV penetration, while meeting the climate targets in each step of the decomposition. This implies that we keep emissions constant throughout the decomposition, so we can compare the steps on purely economic grounds. Fig. 3 summarises this decomposition of impacts in 2050 for GDP results across the three electrification levels.

The *Total* impact represents the overall benefit (in GDP terms) of deploying EVs for climate action, i.e. the higher level of GDP in 2050 compared to a counterfactual, hypothetical scenario, where these EVs are not deployed and the climate target must be met using alternative means. The *Stock* effect reflects the increased expenditures for the purchase of EVs, which puts downward pressure on GDP in the medium and high electrification scenario.^15^ When introducing the assumption of *Learning-by-doing* in batteries, the stock impact is greatly compensated (in fact this is the case as early as 2030), as EVs become cheaper than ICE vehicles over the modelling period. The impact channel labelled *Maintenance* reflects the lower servicing requirements in EVs compared to ICE vehicles, is relatively minor and leads to small GDP gains as the operation costs of vehicles are reduced, freeing up resources for investments and reducing overall mitigation costs. The *Interaction* effect represents the impact on GDP of capturing all three previous assumptions jointly in the CGE model, as these effects reinforce each other. For instance, as vehicles become cheaper to operate, there words, it is additional incentives to purchase vehicles, which in turn leads to higher learning effects in batteries. Finally, the *Fuel Shift* effect, capturing the move away from oil products towards electrification for transport generates GDP gains as lower fuel costs enable firms and households to drive more or spend on other goods and services.^16^ With increasing levels of transport electrification, the costs of mitigation efforts are reduced, leading to increases in output across the economy. The results of the simulations show that alternative electrification pathways for transport can have opposite effects on the costs of climate action. Results for the low-electrification scenario suggest that the moderate adoption of EVs leads to small GDP losses, as the low deployment removes the potential benefits of strong battery costs learning effects, while the fuel shift effect is negative (i.e. reflecting the costs of decarbonising transport through other, more expensive fuels). On the other hand, a high level electrification of transport shows strong positive GDP effects across all channels (net *Stock + Learning* effect, *Maintenance* and *Fuel shift*).

### 3.3 Employment results

**Fig. 4:**
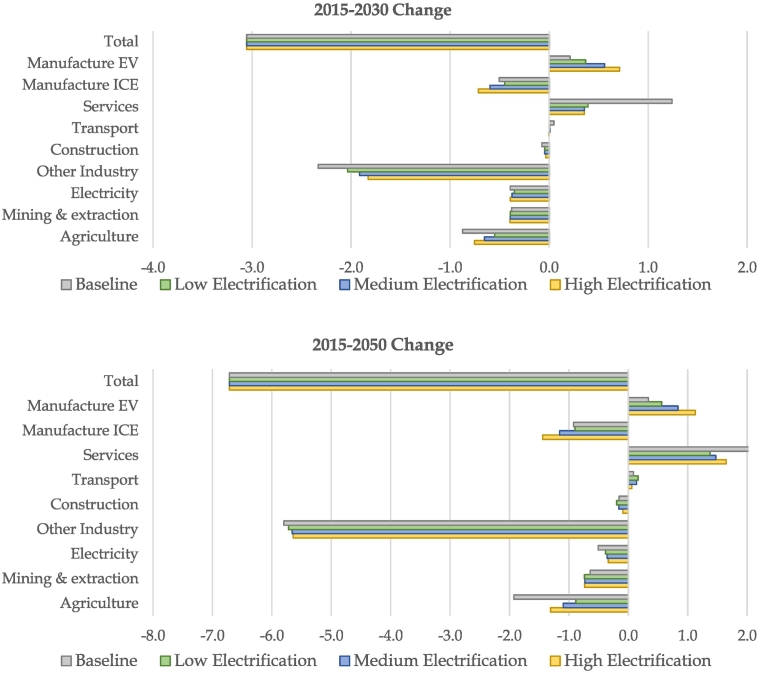
Evolution of sectoral employment in million jobs (EU, between 2015 and 2030 and 2015–2050) Source: JRC-GEM-E3 results (perfect labour market closure, see appendix for more results).

Policies promoting the reduction of GHG emissions as well as the deployment of EVs lead to substantial structural shifts in the EU economy, resulting in an evolution of sectoral employment over the period. Fig. 4 highlights the changes in absolute employment between 2015 and 2030, and 2015 and 2050 in the baseline and in the three electrification scenarios.^17^ In the baseline, total employment reduces by 3.1 % or 6.7 million jobs between 2015 and 2050, reflecting demographic assumptions on the evolution of the labour force (total EU population and aging).

The baseline also illustrates the expected structural shift away from industry and agriculture, towards a more service-based economy for the EU. The increase in employment in services contributes to the sector being by far the largest employer in 2050. The baseline also incorporates some degree of climate action and energy transition, where fossil-fuel supplying sectors exhibit strong employment losses (−65 %). The baseline integrates a conservative trajectory for the deployment of EVs (as explained in Section 2). This translates into a strong increase in employment for the manufacturing of EVs (close to 255 % between 2020 and 2030), to the detriment of employment in manufacturing of traditional internal combustion engine (ICE) vehicles (−28 %).

Under the three electrification scenarios reaching the climate target, the low-carbon trends are further intensified. Stronger employment losses occur in fossil fuel sectors and ICE vehicle manufacturing. In contrast, the climate targets stimulate the sectors supplying low carbon fuels, i.e. electricity generation (largely decarbonised in the scenarios), and agriculture for biofuels. The lower the degree of road transport electrification (low to high), the more positive the impact on agriculture, as decarbonisation must be achieved through more significant shares of sustainable biofuels.

Employment gains in manufacturing of BEVs are two to three times those of the baseline in the medium electrification scenario and high electrification scenario. While manufacturing of conventional combustion engine vehicles experiences employment losses with higher degrees of electrification, the impact on total vehicle manufacturing is a small net increase, driven primarily from costs reductions assumptions over time (learning in batteries and lower maintenance requirements) leading to increases in demand for vehicles.^18^

Similarly, sectors impacted by supply-chain effects from BEV deployment show different behaviour from the baseline. For instance, other industry, which includes battery manufacturers, experiences lower employment reductions, especially in the medium term (2030) as they ramp-up production. The services sector is impacted negatively both by the lower maintenance services requirements of BEVs and reduced total demand due to climate action across the globe depressing GDPs. The relatively large impact in absolute terms of the services sector is primarily due to its relative size compared to other sectors in the model, it effectively acts as a “reservoir” for labour needed in other parts of the economy. Employment in electricity supply increases with further degrees of electrification of road transport but only post-2030, when domestic demand for electricity increases compared to the baseline.

Results above are based on assumptions of perfect labour markets (PLM), where wages are fully flexible to maintain the unemployment rate at the level of the baseline, thus the scenarios above do not result in changes in aggregate employment. We model the same set of scenarios under an imperfect labour market model closure, where involuntary unemployment can result from changes in labour demand, following a wage curve formulation in JRC-GEM-E3. The results are shown in Table 4 for the three climate mitigation scenarios with varying degrees of road transport electrification, both in terms of GDP (a) and employment (b).^19^

**Table 4:** Macroeconomic and employment impacts under alternative labour market closures.

	2030		2050	
	Perfect LM	Imperfect LM	Perfect LM	Imperfect LM
(a) EU 27 + UK GDP - % change from baseline				
Low Electrification	−0.26	−0.44	−0.54	−0.85
Medium Electrification	−0.15	−0.23	−0.38	−0.53
High Electrification	−0.06	−0.06	−0.24	−0.26

(b) EU27 + UK Employment - % change from baseline				
Low Electrification	0.00	−0.39	0.00	−0.46
Medium Electrification	0.00	−0.15	0.00	−0.10
High Electrification	0.00	0.06	0.00	0.15

Source: JRC-GEM-E3 results.

When not assumed fixed, employment experiences a slight decline out to 2050 compared to the baseline, driven by increasing pressure on wages from the transition to a low carbon economy. This is the case for both the low and medium electrification scenarios, where employment falls by 0.46 % and 0.1 % respectively by 2050. In contrast, with higher ambition in terms of road transport electrification, employment is stimulated in the medium and long-term compared to the baseline, as costs of owning and operating vehicles decrease, leading to further increases in output in BEV manufacturing and its supply chain (equipment goods including battery manufacturing, non-ferrous metals, chemicals). The sectoral results under the imperfect labour markets remain qualitatively similar to those presented in Fig. 4, but with more amplitude in changes from the baseline.

### 3.4 Occupations and skills

As the electrification of road transport and decarbonisation will generate significant shifts in the sectoral composition of employment, this will have further implications for the labour market and in particular on the composition of the workforce in terms of occupations and skills. As described in the methods section, we combine CEDEFOP projections of occupations and skills with the sectoral employment results derived under the baseline and the electrification scenarios. The CEDEFOP projections are enhanced to include occupations and skills structures for the manufacturing of BEVs. By using this method, the changes in occupations and skills result from shifts in employment across sectors and EU countries in the various scenarios. Importantly, these shifts do not reflect “within sector” skills and occupations shifts, except for vehicle manufacturing, where we make explicit assumptions about their evolution.

**Fig. 5:**
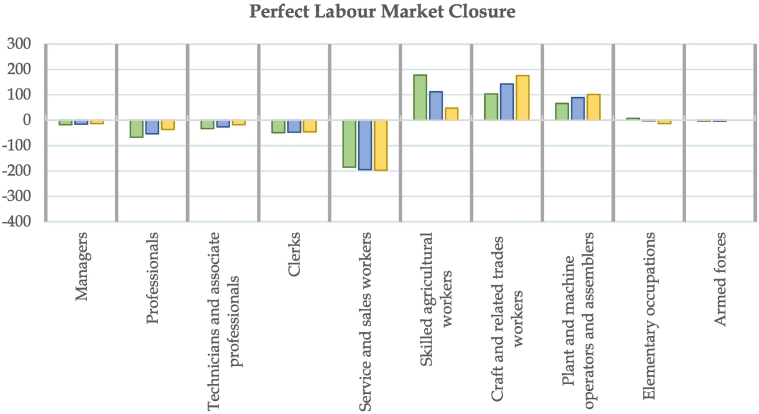
Difference in Employment (000 s jobs) vs. baseline in 2030 by occupation group.

As the CEDEFOP skills forecast is available out to 2030, we restrict our analysis to the same timeframe, using sectoral employment results from the JRC-GEM-E3 in 2030. Fig. 5 presents the main shifts across the nine occupational groups as a result of the three electrification scenarios. Climate action (across all scenarios) will shift employment demand away from certain occupation groups and towards others. Agricultural workers, craft and trade workers, and plant operators and assemblers could benefit from investments in the transition as production of biofuels and renewal of equipment and infrastructure becomes a priority in the next decade to achieve the transition. This is to the detriment of services-based occupations (clerks, sales workers, managers, professionals), more prominent in sectors experiencing employment drops. This effect is particularly pronounced in the case of fixed unemployment (perfect labour market), where labour supply is fixed and must be reallocated across sectors and occupations, in comparison with the ILM closure showed in Appendix B5.

The degree of transport electrification also has a significant impact on allocation of jobs across occupation groups. Lower electrification increases the role of biofuels in reducing emissions in the transport fuel mix, leading to the strongest increase in demand for agricultural workers in 2030 compared to the baseline. In contrast, medium and high levels of electrification reduce the reliance on biofuels and agricultural workers, but boosts demand for science and engineering professionals, as well as technician occupations in vehicle manufacturing, partly driven by the assumptions about the evolution of employment structure within the BEV manufacturing sector. Crafts and related trades workers, as well as plant and machine operators and assemblers are also in higher demand in scenarios with higher electrification of transport, linked to increases in manufacturing sectors outputs. The reduced reliance on these occupations within vehicle manufacturing from the switch towards manufacturing BEVs (in our assumptions) does not compensate for this broader trend in employment.

Similarly, the evolution of skills levels within the labour force will vary across the scenarios driven by the sectoral employment shifts from the model. Table 5 provides a summary of changes in skill level both over the period 2015–2030 in the baseline (left), and as a difference from the baseline in 2030 for each of the three electrification scenario (right). The baseline evolution exhibits a sharp upskilling trend over the next decade in the EU. The strong decrease in low skilled employment (−31.2 % across the economy) is reflected across all sectors. Overall, medium-skilled employment also reduces over the same period by 8.4 %, but to varying degrees across sectors (with for example strong drops in electricity and mining activities, and moderate in transport or services). In contrast, the high skilled group experiences a significant increase in employment by 2030 of 23.9 %, as all sectors are expected to upskill their workforce.

**Table 5:**
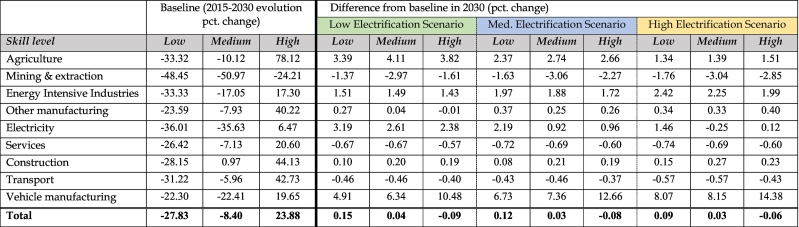
Evolution of employment by skill level in Scenarios with Perfect Labour Market Closure.

This baseline reflects primarily the exogenous inputs from the CEDEFOP forecast, and it is therefore useful to compare how decarbonisation (and the degree of road transport electrification) affects this general projected trend of upskilling. Results across the three scenarios show that the burden of decarbonising the EU economy may not fall disproportionately on low-skilled workers, but on the contrary could slightly moderate the upskilling trend observed in the baseline. In Table 5 (right), we focus on the results of the scenarios where total employment supply is fixed (perfect labour market), but the relative changes in skill composition across the scenarios described below also hold under the imperfect labour market closure (see Appendix Table B.5). In all scenarios, aggregate employment changes by skill level are very small. We see a slight increase in low-skilled and medium-skilled labour compared to the baseline, and a small decrease in high-skill labour. This impact is primarily the result of employment shifts towards sectors with a relatively lower skilled workforce such as agriculture, construction or manufacturing.

The sector experiencing the largest impact in terms of skills is logically the manufacturing of vehicles. Despite increases in employment in all skill levels due to the increased demand for vehicles, there is a clear upskilling trend associated with higher penetration of BEVs in the fleet through the assumptions made about the BEV manufacturing sector compared to the existing ICE manufacturing sector. In other sectors, increased electrification results in lower impacts on skill structure. In agriculture, decarbonisation entails increases in employment at all skill levels, but with lower electrification, there is more reliance on medium-skill labour relative to others. In electricity, employment reduces with increasing degrees of electrification, but this is a transitional effect which is due to presenting skill and occupation results only to 2030. In fact, the shift towards electrification reflects both a move from fossil fuels to electricity but also general efficiency gains in terms of primary energy. Only post-2030 does EU demand for electricity increase, and alongside demand for employment in the electricity sector.

Overall, the small variations in skill composition in the aggregate results suggest that more focus should be placed on investigating shifts in skills and occupations within the different sectors important to the low-carbon transition (for instance those contributing to energy efficiency improvements in buildings, the deployment of renewables, the transition to low-carbon mobility, etc.).

## 4 Conclusions and policy implications

The electrification of road transport is accelerating across the globe, with significant increases in electric vehicle market shares in China, Europe and the United States in particular (IEA, 2020c). While long-term projections on the number of EVs in the car and truck fleets vary across models and methodologies, the technology is widely expected to make up the majority of the total vehicle stock by mid-century, as a result of increasingly stringent climate policies and technology advancements, notably in batteries. Such fleet transformation in the coming three decades will be associated with large shifts in the manufacturing of motor vehicles and their supply-chain in the near-term, as well as changes in the operation of vehicles (fuel shifting, energy efficiency, maintenance schedules, etc.).

This paper explores the macroeconomic implications of road transport electrification as a contributing policy to reaching Europe's climate targets. We combine techno-economic assumptions about the composition and operation of the fleet with fuel consumption projections from energy system models into a set of climate mitigation scenarios with varying degrees of road transport electrification. We run these scenarios in an augmented version of the JRC-GEM-E3 model, which explicitly represents the manufacturing of BEVs as a separate economic activity from the manufacturing of conventional ICE vehicles. This new methodology allows for a more detailed analysis of macroeconomic impacts in two major ways: first the supply chain implications of the deployment of EVs can be more clearly identified than if all vehicles were produced using a single input structure. This has large implications in terms of employment, and therefore on occupations and skills. Second, this also allows for a new decomposition analysis, where the various channels of impacts from EVs on macroeconomic results can be explored. Our modelling suggests that a higher degree of road transport electrification can contribute to reducing the costs of climate mitigation policies. The GDP impact for EU27 + UK of implementing policies to limit climate change to 2 °C range from −0.54 % in 2050 in a low-electrification scenario to −0.24 % in a high electrification scenario, compared to a baseline of limited climate action. This range extends from −0.85 % to −0.26 % when relaxing assumptions about the labour market, and allowing labour supply to adjust to real wage changes. The employment results under this labour market closure also suggest that decarbonisation through high electrification of road transport can actually stimulate employment at aggregate level in the EU, through increased economic activity. However, this result is partly contingent on an increased demand for vehicles resulting from this increased activity, and whether the assumption made in the modelling of maintaining current supply-chain locations for vehicle manufacturing hold true in the race towards electric vehicle and battery production. These initial results suggest the importance of pursuing an ambitious EU industrial strategy in order to maintain and develop the EU's competitive position in the automotive and battery markets, in line with, for example, the European Battery Alliance launched in 2017.^20^ Further insights could be gained from varying assumptions about uptake of EVs around the world and evolving value chains for new players exporting to the EU like China could be further explored in new scenarios. The analysis could be further enhanced by considering the associated investments in charging infrastructure, which are likely to be substantial. EU estimates suggest annual investment of 0.6–1.2 €bn between 2021 and 2030 and 2.0–2.4 €bn between 2031 and 2050 for electric charging points depending on the level of ambition (European Commission, 2021, European Commission, 2021). A similar approach to this paper using bottom-up data to derive costs of electrification infrastructure could be used to extend the study of macroeconomic impacts. Likewise, further research should include considerations of potential impacts of supply-chain issues in critical raw materials or components on EV deployments and costs.

One novelty of the modelling exercise is the ability to distinguish impacts of each of the various elements of fleet electrification. We highlight that the positive impacts of higher road transport electrification for climate action by 2050 are primarily driven by the reduced costs BEVs entail compared to their ICE counterfactual over time (reduced maintenance requirements and lower fuel costs, and in particular how the higher upfront purchasing costs of BEVs in the base year of the modelling can be offset by reductions in battery costs over the medium-term). EV deployment has accelerated across the globe, and climate and transport emission reduction policies have been made increasingly stringent. The mechanisms highlighted in this work may therefore become increasingly relevant as EVs rapidly gain market share. Once again, these results are contingent on strong reductions in prices of BEV, which ceteris paribus, can be linked to reductions in battery costs. However, further exploration of the role of raw materials costs in EV costs is warranted, in a context of complex global value chains, to further contribute to the EU's critical raw material policies.

Furthermore, while ambitious policies in favour of road transport electrification could help the EU achieve its climate goals at lower costs, they will affect the future composition of the workforce. This paper proposed a new methodology to extend the macroeconomic analysis of electrification policies applying exogenous projections of EU occupations and skills to the sectoral employment results of the JRC-GEM-E3 model. The shift from conventional vehicles towards BEVs affects not only workers in vehicle manufacturing, but also more broadly in sectors that are complements (batteries, electricity supply) and substitutes (fossil fuel production, biofuel production) to EV deployment. These economy-wide interactions also play a role in shaping the structure of occupational demand in the coming years and decades. The findings of this paper confirm the importance of developing flexible sector-specific analyses and policies to reduce skill mismatch in the energy transition. In particular, in the vehicle manufacturing sector, the recent EU's Skill Partnership for the automotive ecosystem^21^ is a good example of a tailored approach to bridge the gaps highlighted in this study.

While this study expands the set of indicators to cover occupations, skills and tasks, it should be seen as a first step from an aggregate to a more refined assessment of labour market dynamics. Future work should aim to shed light on other potentially important within-sector shifts which were not captured here. Other extensions should also consider the potential longer-term impacts on occupation and skills once the transition towards decarbonised road transport is achieved, and may consider barriers to labour mobility and costs of re-skilling.

## CRediT authorship contribution statement

**Marie Tamba:** Conceptualization, Methodology, Software, Formal analysis, Investigation, Writing – original draft, Visualization. **Jette Krause:** Conceptualization, Methodology, Formal analysis, Investigation, Writing – review & editing, Project administration. **Matthias Weitzel:** Software, Validation, Formal analysis, Writing – review & editing. **Raileanu Ioan:** Investigation, Data curation. **Louison Duboz:** Conceptualization, Formal analysis, Writing – review & editing. **Monica Grosso:** Conceptualization, Writing – review & editing. **Toon Vandyck:** Visualization, Writing – review & editing.

## Figures and Tables

**Table:** 

	JRC-GEM-E3 sector names
1	Crops
2	Coal
3	Crude oil
4	Oil
5	Gas
6	Electricity transmission & distribution
7	Ferrous metals
8	Non-ferrous metals
9	Chemical products
10	Paper products
11	Non-metallic minerals
12	Electric goods
13	Transport equipment
14	Other equipment goods
15	Consumer goods industries
16	Construction
17	Air transport
18	Land transport
19	Water transport
20	Market services
21	Non market services
22	Coal fired power generation
23	Oil fired power generation
24	Gas fired power generation
25	Nuclear generation
26	Biomass generation
27	Hydroelectric generation
28	Wind power generation
29	PV power generation
30	Livestock
31	Forestry
32	Manufacture of motor vehicles
33	Manufacture of electric vehicles

**Fig. B.4:**
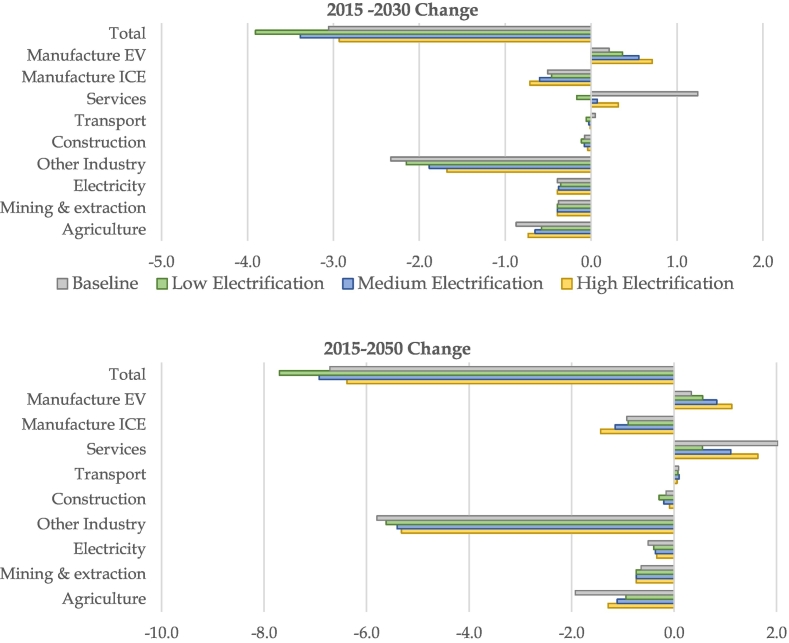
Evolution of sectoral employment in million jobs (EU, between 2015 and 2030 and 2015–2050).

**Fig. B.5:**
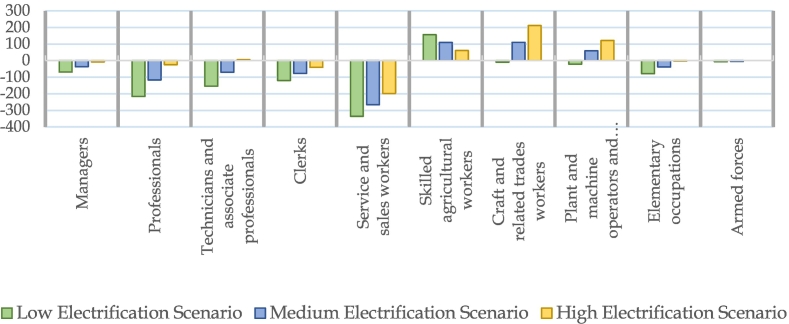
Difference in Employment (000 s jobs) vs. baseline in 2030 by occupation group (Imperfect Labour Market).

**Table B.5:**
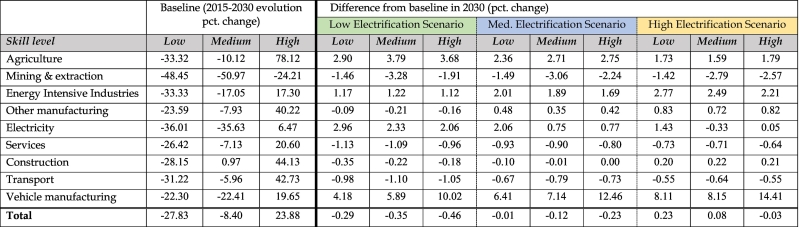
Evolution of employment by skill level in Scenarios with Imperfect Labour Market Closure.

**Table:** 

Occupations – results reporting groups		CEDEFOP	
		Code	Label
0	Armed forces	1	Armed forces
1	Managers	2	Chief executives, senior officials and legislators
		3	Administrative and commercial managers
		4	Production and specialised services managers
		5	Hospitality, retail and other services managers
2	Professionals	6	Science and engineering professionals
		7	Health professionals
		8	Teaching professionals
		9	Business and administration professionals
		10	Information and communications technology professionals
		11	Legal, social and cultural professionals
3	Technicians and associate professionals	12	Science and engineering associate professionals
		13	Health associate professionals
		14	Business and administration associate professionals
		15	Legal, social, cultural and related associate professionals
		16	Information and communications technicians
4	Clerks	17	General and keyboard clerks
		18	Customer services clerks
		19	Numerical and material recording clerks
		20	Other clerical support workers
5	Service and sales workers	21	Personal service workers
		22	Sales workers
		23	Personal care workers
		24	Protective services workers
6	Skilled agricultural workers	25	Market-oriented skilled agricultural workers
		26	Market-oriented skilled forestry, fishery and hunting workers
		27	Subsistence farmers, fishers, hunters and gatherers
7	Craft and related trades workers	28	Building and related trades workers, excluding electricians
		29	Metal, machinery and related trades workers
		30	Handicraft and printing workers
		31	Electrical and electronic trades workers
		32	Food processing, wood working, garment and other craft and related trades
8	Plant and machine operators and assemblers	33	Stationary plant and machine operators
		34	Assemblers
		35	Drivers and mobile plant operators
9	Elementary occupations	36	Cleaners and helpers
		37	Agricultural, forestry and fishery labourers
		38	Labourers in mining, construction, manufacturing and transport
		39	Food preparation assistants
		40	Street and related sales and service workers
		41	Refuse workers and other elementary workers

## Footnotes

1 Regulation ((EU) 2021/1119) adopted by European Council and European Parliament on 9 July 2021, http://data.europa.eu/eli/reg/2021/1119/oj

2 Model documentation can be found at: https://ec.europa.eu/jrc/en/gem-e3

3 Based on the GTAP sectoral disaggregation, the JRC-GEM-E3 sector of manufacture of motor vehicles corresponds 1-to-1 to the GTAP sector mvh - Motor vehicles and parts (NACE C29). Similarly, the JRC-GEM-E3 sector “Other transport equipment” corresponds 1-to-1 to GTAP sector otn “Transport equipment n.e.c” (NACE C30).

4 Only battery and plug-in hybrid vehicles are considered. We do not consider fuel-cell EVs in the present analysis as their production and maintenance would differ from BEVs.

5 The paper focuses on EU27 + UK, as the analysis is based on modelling scenarios derived for the EU's 2050 long-term strategy, adopted while the UK was still a Member of the European Union (https://ec.europa.eu/clima/policies/strategies/2050_en).

6 https://www.renault.it/veicoli/veicoli-commerciali/kangoo-express.html (price) (Last accessed on 20/02/2019)

7 https://www.renault.it/veicoli/auto-elettriche-e-veicoli-elettrici/master-ze.html (price) (Last accessed on 20/02/2019)

8 For the following vehicle types making up more than 95 % of the fleet: small car, medium car, large car, SUV, and light commercial vehicles.

9 We do not explicitly model the manufacturing of hybrid EVs (HEVs), but include these vehicles in the deployment scenarios them implicitly in the modelling through a simplistic assumption they are partly manufactured by each vehicle sector.

10 An increased penetration of electric cars among households might lead to a lower use of public transport once EVs are less costly to own than ICE vehicles, although the corresponding reduced costs of public transport, which increasingly rely on EVs, could mitigate this effect.

11 BNEF (2021) Hitting the EV Inflection Point https://www.transportenvironment.org/sites/te/files/publications/2021_05_05_Electric_vehicle_price_parity_and_adoption_in_Europe_Final.pdf

12 UBS (2017) UBS Evidence Lab Electric Car Teardown –Disruption Ahead? https://neo.ubs.com/shared/d1wkuDlEbYPjF/

13 Where upskilling is defined as “the process of learning new skills or of teaching workers new skills”, while deskilling is defined as “to reduce the amount of skill that someone needs to do a particular job” (Cambridge dictionary, accessed 09 June 2021)

14 The Skills Forecast is developed using a modelling exercise based on assumptions from the EU Reference Scenario 2016, capturing the existing energy, climate and transport policies at the time of publication: https://ec.europa.eu/energy/data-analysis/energy-modelling/eu-reference-scenario-2016_en)

15 In the low-electrification scenario, the lower penetration of EVs in trucks and public transport vehicles has the inverse impact.

16 The fuel shift effect captures the difference between electrifying road transport according to the three energy model scenarios and a hypothetical scenario replacing electricity with other decarbonised fuels, namely biofuels.

17 Results of scenarios under perfect labour market assumption, i.e. no change in total employment compared to the baseline. Results under alternative market closure are presented in Appendix.

18 Despite the assumption in the production structure of BEVs that it requires the same value-added (labour and capital) in value per unit of production as a conventional ICE vehicle.

19 Sectoral results with imperfect labour market closure are presented in Appendix B.

20 The European Battery Alliance (EBA) was launched in 2017 to develop an innovative, competitive and sustainable battery value chain in Europe. https://ec.europa.eu/growth/industry/strategy/industrial-alliances/european-battery-alliance_en

21 The Skills Partnership for the automotive ecosystem, launched in November 2020, aims to bring EU stakeholders together to collaborate on up- and re-skilling workers in the sector (https://www.acea.auto/news/skills-partnership-for-the-automotive-ecosystem/)
